# Supplementary material for: Factors Determining the Functional State of Cardiac Surgery Patients with Complicated Postoperative Period
Source: Int J Environ Res Public Health. 2022 Apr 4;19(7):4329. doi: 10.3390/ijerph19074329 (PMC8998976; doi:10.3390/ijerph19074329)
Supplement: Supplementary file 1 [file ijerph-19-04329-s001.zip › ijerph-1552229-supplementary.pdf]

Suppl. Table S1. Multiple linear regression (Forward) in assessing the distance 6MWT at discharge with perioperative indicators in patients with complications after cardiac surgery (all patients; Model Summary)

Adjusted R Square	Std. Error of the Estimate
,392	64,27934
,514	57,47331
,671	47,31268
,733	42,60734

a. Predictors: (Constant), 6MWT after ICU

b. Predictors: (Constant), 6MWT after ICU, Cardiopulmonary bypass duration

c. Predictors: (Constant), 6MWT after ICU, Cardiopulmonary bypass duration, Body mass index

d. Predictors: (Constant), 6MWT after ICU, Cardiopulmonary bypass duration, Body mass index, Left foot extensor strength

6MWT – six minute walk test; ICU - Intensive Care Unit

Suppl. Table S2. Multiple linear regression (Forward) in assessing the distance 6MWT at discharge with perioperative indicators in patients with complications after cardiac surgery (patients with NMES; Model Summary)

Model	R	R Square	Adjusted R Square	Std. Error of the Estimate
1	,738[a]	,545	,504	58,96005
2	,845[b]	,714	,657	49,03027

a. Predictors: (Constant), age

b. Predictors: (Constant), age, Cardiopulmonary bypass duration

Suppl. Table S3. Multiple linear regression (Forward) in assessing the distance 6MWT at discharge with perioperative indicators in patients with complications after cardiac surgery (patients without NMES; Model Summary)

Model	R	R Square	Adjusted R Square	Std. Error of the Estimate
1	,756[a]	,572	,536	53,64813
2	,889[b]	,790	,752	39,25813
3	,928[c]	,862	,820	33,40927

a. Predictors: (Constant), Body mass index

b. Predictors: (Constant), Body mass index, 6MWT after ICU

c. Predictors: (Constant), Body mass index, 6MWT after ICU, Left foot extensor strength
